# Deep Brain Stimulation of the Medial Septal Nucleus Induces Expression of a Virally Delivered Reporter Gene in Dentate Gyrus

**DOI:** 10.3389/fnins.2020.00463

**Published:** 2020-05-12

**Authors:** Anton Fomenko, Darrin J. Lee, Chris McKinnon, Eun Jung Lee, Mitchell L. de Snoo, Elise Gondard, Clemens Neudorfer, Clement Hamani, Andres M. Lozano, Lorraine V. Kalia, Suneil K. Kalia

**Affiliations:** ^1^Krembil Research Institute, University Health Network, Toronto, ON, Canada; ^2^Department of Neurological Surgery and USC Neurorestoration Center, Keck School of Medicine of USC, Los Angeles, CA, United States; ^3^Division of Neurosurgery, Sunnybrook Health Sciences Centre, University of Toronto, Toronto, ON, Canada; ^4^Division of Neurosurgery, Department of Surgery, Toronto Western Hospital, University of Toronto, Toronto, ON, Canada

**Keywords:** neuromodulation, deep brain stimulation, viral vector, gene expression, medial septal nucleus, hippocampus

## Abstract

**Background:**

Mechanisms of deep brain stimulation (DBS) remain controversial, and spatiotemporal control of brain-wide circuits remains elusive. Adeno-associated viral (AAV) vectors have emerged as vehicles for spatiotemporal expression of exogenous transgenes in several tissues, including specific nuclei in the brain. Coupling DBS with viral vectors to modulate exogenous transgene expression remains unexplored.

**Objective:**

This study examines whether DBS of the medial septal nucleus (MSN) can regulate gene expression of AAV-transduced neurons in a brain region anatomically remote from the stimulation target: the hippocampal dentate gyrus.

**Methods:**

Rats underwent unilateral hippocampal injection of an AAV vector with c-Fos promoter-driven expression of TdTomato (TdT), followed by MSN electrode implantation. Rodents received no stimulation, 7.7 Hz (theta), or 130 Hz (gamma) DBS for 1 h one week after surgery. In a repeat stimulation experiment, rodents received either no stimulation, or two 1 h MSN DBS over 2 weeks.

**Results:**

No significant differences in hippocampal TdT expression between controls and acute MSN DBS were found. With repeat DBS we found c-Fos protein expression was induced and we could detect increased TdT with either gamma or theta stimulation.

**Conclusion:**

We demonstrate that viral vector-mediated gene expression can be regulated spatially and temporally using DBS. Control of gene expression by DBS warrants further investigation into stimulation-responsive promoters for clinical applications.

## Introduction

Deep brain stimulation (DBS) is a standard treatment for medication-refractory movement disorders such as Parkinson’s disease and essential tremor (Kalia et al., 2013; Lozano et al., 2017). Electrical fields induced by DBS can directly modulate the activity of target neurons as well as indirectly affect distant neuronal populations within the same circuitry to alter electrical oscillations of brain networks (Lozano et al., 2017). Previous studies have demonstrated that DBS also modulates endogenous gene expression at the stimulation target, as well as in distant synaptically-connected brain regions (Gondard et al., 2015; Mann et al., 2017). Recently, adeno-associated viral (AAV) vectors have emerged as vehicles for robust spatiotemporal expression of exogenous transgenes of interest in several tissues, including the brain (Hudry and Vandenberghe, 2019). After injection into brain tissue, AAVs are able to induce long-term expression of desired genes in both neuronal and non-neuronal cells largely dictated by viral serotype (Taymans et al., 2007; Mitchell et al., 2010; Aschauer et al., 2013). Despite the potential benefits of AAVs as a modulatory tool of the downstream effects of DBS, this application has remained unexplored.

Several studies provide electrophysiologic evidence that DBS of the medial septal nucleus (MSN) has modulatory effects on neurons in distally-connected regions, such as the hippocampus. For instance, DBS of the medial septum within an electrically-stimulated rodent septohippocampal preparation showed that glutamatergic neurons within the MSN synaptically drive hippocampal pyramidal cells, and elicit rhythmic firing of CA3 cells at theta frequencies (Huh et al., 2010). *In vivo* evidence also suggests that electrolytic lesions in the rodent MSN reduce intrinsic hippocampal theta rhythm and impair spatial memory, suggesting an important modulatory rode of the MSN (Winson, 1978).

Elevated expression of c-Fos, an immediate early gene, by neurons can be used as a marker of recent increased neuronal activity, especially when induced by DBS (Kovács, 2008; Gondard et al., 2015). Our (Gondard et al., 2015) and other groups have used c-Fos as a robust functional marker of brain regions undergoing changes in electrical activity in response to DBS or neuroactive drugs. The synthesis of c-Fos protein after mRNA expression, and its turnover occurs with a 2-h half-life, with decay in expression afterward, peaking between 1 and 6 h after stimulation, depending on the brain region (Müller et al., 1984; Schulte et al., 2006; Saryyeva et al., 2011). Although c-Fos, like other immediate-early genes, also functions as a transcription factor and regulator of downstream target genes, c-fos and other immediate-early genes serve as useful and reliable indicators of site-specific neural activation in preclinical models (Torres-Sanchez et al., 2017).

Here, we sought to investigate upregulation of the expression of a reporter gene by DBS in AAV transduced neurons in a brain region anatomically remote from the stimulation target. Specifically, we examined whether MSN DBS could induce reporter gene expression under the control of the c-Fos promoter in a synaptically-connected site, the dentate gyrus (DG). We investigated the effect of two physiologically-relevant stimulation frequencies, theta and gamma, and two stimulation paradigms, acute and repeated, on the expression of a fluorescent c-Fos reporter in the DG.

## Methods

### Animals

The study protocol was institutionally approved in accordance with the Canadian Council on Animal Care guidelines. Forty-two adult female Sprague-Dawley rats (12–14 weeks of age, weight 280–320 g) were housed under a 12-h light/dark cycle with continuous access to standard rat chow and water. For the acute stimulation experiment, animals were randomized to one of the following 1-h stimulation groups: no stimulation (sham, 5 animals), theta (7.7 Hz, 6 animals), or gamma (130 Hz, 6 animals) DBS. In the repeated stimulation experiments, animals were randomized into the following groups, where they were stimulated for 1 h, once a week, for 2 weeks: sham (7 animals), theta (10 animals), and gamma (8 animals). Animals numbers are reflective of final animal numbers used after exclusion of mistargeted electrodes. During surgical interventions, animals were anesthetized with 2–4% isoflurane and fixed in a stereotactic frame (KOPF, Model 902), and procedures were performed under aseptic conditions.

### AAV Injection Surgery

**FIGURE 1:**
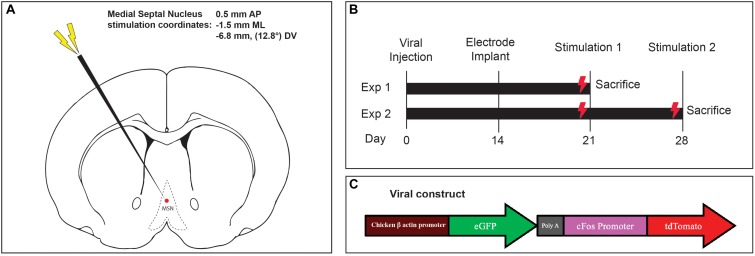
**(A)** Medial septal nucleus DBS electrode location overlaid on stereotactic rat atlas. **(B)** Experimental design and stimulation timeline. **(C)** Schematic of the custom AAV5-c-Fos construct used.

After shaving and incising the scalp, burr holes were made in the right skull over the hippocampus (−3.3 mm AP, −2.0 mm ML) and striatum (0 mm AP, −3.0 mm ML) (Paxinos and Watson, 1997) with an electric drill. The dura was punctured and the tip of a 10 μL glass microsyringe (Hamilton Company, Reno, Nevada) was inserted into the dentate gyrus (−3.4 mm DV) and striatum (−4.0 mm DV). At each location, 2.0 μL (2 × 10^11^ genome copies/mL) of AAV-5 containing a vector with green fluorescent protein (GFP) under control of the chicken beta actin promoter and tdTomato (TdT) under control of the c-Fos promoter (Figure 1C) was injected over 4 min at each target. The microsyringe was gently withdrawn, the scalp wound was closed with surgical clips, and the animals were awoken.

### Electrode Implantation Surgery

Fourteen days later, the incision was reopened, a frontal burrhole was made (0.5 mm AP, -1.5 mm ML), and a twisted bipolar stimulating electrode 0.127 mm in diameter (Plastics One Inc, Raonoke, Virginia) was implanted into the MSN, -6.8 mm deep at 12.8 degrees (Figure 1A). Four precision screws (JJ Morris Co, Southbridge, Massachusetts) were superficially inserted into the skull and a self-curing methylmethacrylate headcap (Jet Denture Powder and Liquid) was molded to secure the electrode to the skull. The animals were allowed to wake and recover in their homecage.

### Deep Brain Stimulation Protocol

Two experiments were conducted to assess the effects of one-time acute MSN DBS, vs. repeated stimulation. In the single stimulation experiment, 1 week after electrode implantation, rodents received either no stimulation, theta (7.7 Hz) or gamma (130 Hz) MSN DBS for 1 h (Figure 1B). Animals were euthanized 1.5 h after completing stimulation. In the repeat stimulation experiment, rodents received either no DBS, 7.7 or 130 Hz MSN DBS for 1 h one week after implantation, and again at 2 weeks after implantation followed by euthanasia 1.5 h after the final stimulation (Figure 1B). An isolated STG 4004 stimulator (Multichannel Systems, Reutlingen, Germany) delivered electrical stimulation with a 100 μs pulse width and 80 μA current while the animal was freely roaming in its home cage (Gondard et al., 2015).

### Histology

**FIGURE 2:**
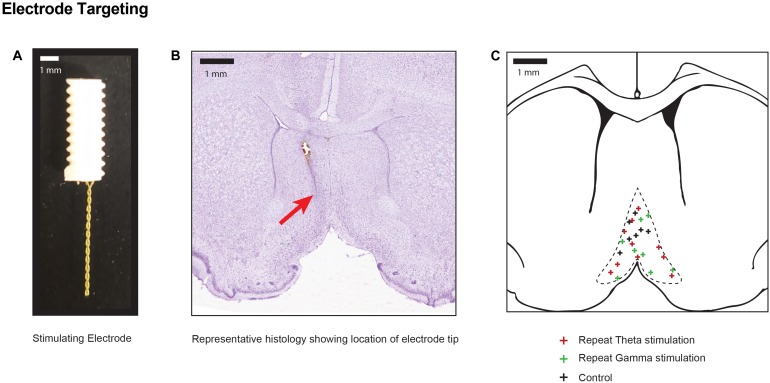
Electrode positions within stimulation target **(A)** Photograph of the two-channel bipolar twisted electrode used for DBS of the medial septal nucleus. **(B)** Representative cresyl violet histology specimen at the level of the medial septum (0.5 mm AP) indicating the final position of the electrode tip in one animal with a red arrow. **(C)** Pooled location of electrode targets within the MSN for sham controls, and repeated theta and gamma stimulation.

As previously described, 1.5 h after completion of DBS, animals were euthanized under deep isoflurane anesthesia by transcardiac perfusion with 200 mL of heparinized 0.9% saline (Gondard et al., 2015). The electrode was carefully withdrawn, and the brain was removed and immediately fixed with 4% paraformaldehyde and cryoprotected in 30% sucrose. The brain was cut into 40 μm coronal sections from +1.0 to -4.80 mm. Sections with visible electrode tracts were mounted and stained with cresyl violet to identify electrode tip placement (Figure 2). Animals with electrode tips outside the predefined area of the MSN were excluded (Paxinos and Watson, 1997). Every fifth hippocampal and striatal section were mounted and scanned on LSM700 confocal microscope (Carl Zeiss Microscopy LLC, Thornwood, New York) and captured in two channels: excitation λ = 561 nm (TdT) and 488 nm (GFP).

### Image Analysis and Quantification

**FIGURE 3:**
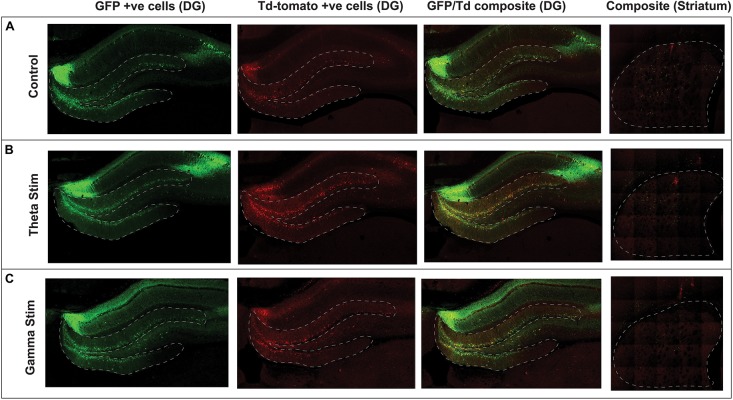
Confocal immunofluorescence imaging of representative hippocampal dentate gyrus and striatum adjacent to the virus injection site. Native fluorescence from the repeat stimulation experiment is shown in the three stimulation groups: **(A)** Control (no stimulation) **(B)** Theta stimulation **(C)** Gamma frequency stimulation. The green GFP channel (green) serves as a reporter for transduced hippocampal neurons, whereas the Td Tomato channel (red) shows the transduced cells with concomitant c-Fos expression.

For quantification, the location of the injected virus, and one section anterior (+200 μm) and one posterior (-200 μm) was chosen, as virus expression was most consistent within this region (Figure 3). The DG and striatum were outlined manually based on the rat stereotaxic atlas (Paxinos and Watson, 1997). A semi-automated quantification algorithm using the HALO Image Quantification Suite (Indica Labs, Albuquerque, New Mexico) was used to quantify GFP and TdT positive cells within the slices (Yousef et al., 2017). Raw data was tabulated in a spreadsheet and a ratio of co-labeled GFP-positive and TdT-positive neurons to all GFP-positive cells in the DG and striatum: (GFP+ and TdT+)/GFP+ was calculated for each animal’s representative dentate gyrus and striatal sections. Separate sections from the hippocampus were stained with a primary 9F6 Rabbit c-Fos antibody (New England Biolabs, Ipswich, Massachusetts) and a fluorescent secondary antibody, Goat anti-Rabbit IgG (H+L) Alexa Fluor 488 (Thermo Fisher Scientific, Waltham, MA, United States) (Gondard et al., 2015). Sections were mounted in fluorescence medium and number of cells with endogenously expressed c-Fos were quantified with the HALO image analysis software within the same three sections.

## Results

**FIGURE 4:**
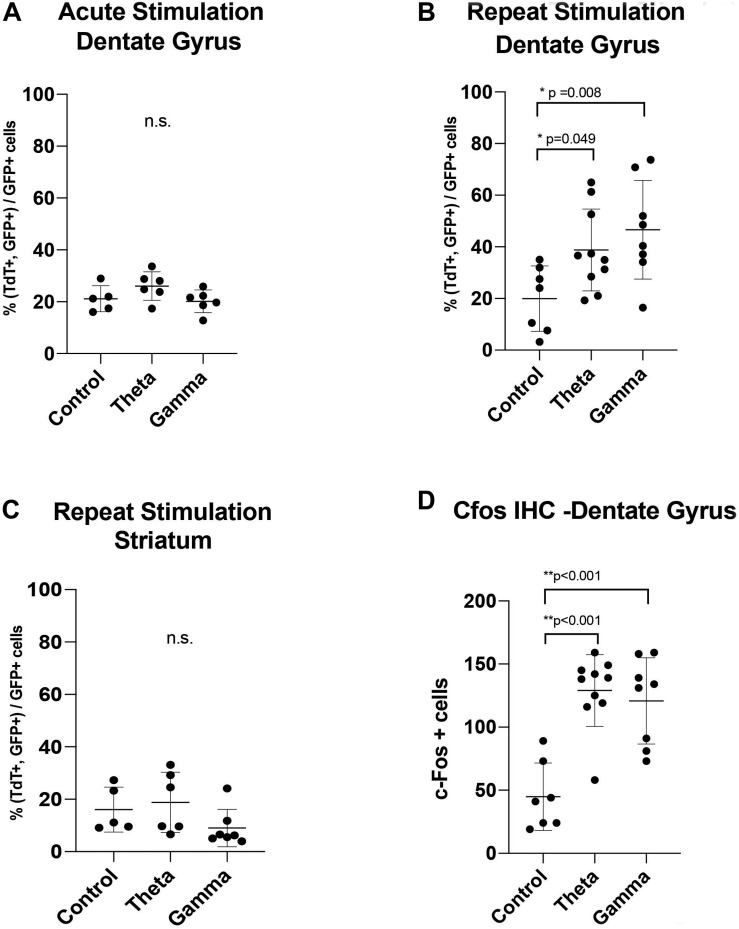
Graphs showing the results of: **(A)** Acute one-time stimulation, showing no significant difference in percentage of c-Fos driven fluorescent reporter (TdTom) positive cells between groups. **(B)** Repeated stimulation, showing significantly increased percentage of colabeled c-Fos+GFP reporter expression with theta and gamma stimulation, compared to unstimulated controls. **(C)** Negative spatial control, showing no difference in stimulation groups in percentage of colabeled c-Fos+GFP fluorescent reporter expression at a synaptically unconnected site, the striatum **(D)** Immunohistochemistry of endogenous c-Fos expression at the dentate gyrus within the hippocampus, showing a significant rise after theta and gamma repeat stimulation. Data points are animals with correctly-targeted electrodes. Whisker plots represent mean with standard deviation. *P*-values are given for Dunnett’s multiple comparisons, when ANOVA is significant (*p < 0.05, **p < 0.005).

After AAV gene delivery into the dentate gyrus of the hippocampus, we detected reporter GFP expression widely distributed in neurons within the DG (Figure 3). We first tested whether single 1 h MSN DBS at theta frequency (7.7 Hz) or gamma frequency (130 Hz) could induce c-Fos promoter driven expression of the TdT gene following AAV gene delivery to the DG. Using this single 1 h stimulation paradigm we found no difference (*p* = 0.1308, ANOVA) in percentage of dentate gyrus TdT positive cells out of constitutively expressive GFP cells, among unstimulated controls, 7.7 Hz DBS, or 130 Hz DBS 1 week after electrode implantation (Figure 4).

Previous work by our group and others showed that multiple electrical stimulation sessions produce the most robust expression of markers of neural activity (Gondard et al., 2015; Mann et al., 2017). Therefore, we next tested whether repeat MSN DBS was necessary to induce gene expression in the DG, we applied 1-h MSN stimulation twice over 2 weeks. Repeat stimulation resulted in a significant increase (*p* = 0.0133, ANOVA) in the percentage of TdT positive cells out of GFP-expressing cells, with either 7.7 Hz (Mean difference 18.81 SE ± 7.99 *p* = 0.0498, Dunnett’s test) or 130 Hz (Mean difference 26.66 ± 8.387, *p* = 0.0081, Dunnett’s test) stimulation compared to no stimulation (Figure 4). In our experiment, AAV delivery and cell transduction was consistent across all conditions within the quantified dentate gyrus sections. Lastly, we confirmed immunohistochemistry of the DG showed significantly elevated endogenous c-Fos signal in the 7.7 and 130 Hz stimulated animals (*p* < 0.001, ANOVA) compared to unstimulated controls (Figure 4).

To confirm that these findings were specific to stimulation of the septohippocampal circuit that underwent DBS, we next examined whether AAVs directly injected into the striatum, a synaptically remote target, could also be activated by MSN stimulation. Using the repeated stimulation paradigm we found that the number of TdT positive cells in the striatum, a structure outside the targeted circuit, was not significantly different (*p* = 0.2973, ANOVA) between animals treated with no stimulation (5 animals), repeat theta stimulation (6 animals) and repeat gamma stimulation (7 animals) (Figures 3, 4).

## Discussion

Here, we demonstrate that expression of virally delivered genes can be induced using repeated low-frequency theta (7.7 Hz) and high-frequency gamma (130 Hz) DBS to a target along a circuit. We investigated the c-Fos promoter driven expression of a reporter gene (TdT) in this proof-of-principle study since c-Fos is an immediate-early marker of neural activation and synaptic activity (Saryyeva et al., 2011). We found that the rise in gene expression within the DG increased by 19% after repeat theta stimulation, and 27% after repeat gamma stimulation, compared to the sham group. Since hippocampal theta rhythm is thought to be mediated via medial septal pacemaker cells, we had initially hypothesized that 7.7 Hz DBS of the MS would have preferentially entrained neural activity, compared to 130 Hz stimulation (Buzsáki and Moser, 2013). Nevertheless, gamma oscillations in the dentate gyrus of freely behaving rat are also tightly linked to memory consolidation and are often entrained with theta rhythm, which could explain why both frequencies were successful (Chrobak and Buzsáki, 2018). 7.7 and 130 Hz stimulation both resulted in increased gene expression, suggesting that gene expression can be activated by stimulating the circuit parameters that can affect the excitability of dentate gyrus neurons to altered septal neuron spiking activity.

Previous work by our group found that chronic 130 Hz DBS of the anterior fornix within the Papez circuit induces robust endogenous c-Fos expression in the rat hippocampus, which correlates with the immunohistochemistry results we obtained with MSN stimulation (Gondard et al., 2015). Though literature suggests that a single stimulation timepoint is often sufficient to elevate early-gene expression in the brain region surrounding the electrode, the same is not clearly established for other distant, synaptically-connected sites. Indeed, stimulation of one target can sometimes also cause paradoxical (decreased) c-Fos expression in brain regions within a common circuit, but distant from the electrode, as seen other animal literature (Ewing et al., 2013; Huguet et al., 2017). Since we stimulated the medial septum and analyzed a specific portion of the hippocampus (dentate gyrus), our finding of elevated c-Fos only after repeat stimulation may reflect a more complex interplay of MSN-regulation of hippocampal theta rhythm, or a facilitated c-Fos expression in distant brain regions, as seen in other studies (Shijo et al., 2008).

We now provide evidence that we can utilize DBS and intrinsic neuronal circuitry to induce expression of a viral reporter-driven exogenous protein at a distant target. AAV-mediated c-Fos expression in the striatum, a distant and synaptically unrelated target, was not significantly different between control and stimulation groups. Though we observed a non-significant increase in mean percentage of TdT positive cells out of GFP-expressing cells after theta stimulation, and a non-significant decrease after gamma stimulation, the mean differences (-2.7 and +7.05%, respectively) were relatively small and likely due to inter-animal variability in c-Fos expression. Since the striatum is not directly synaptically connected to the septohippocampal circuit, our findings suggest that this paradigm is amenable to circuit-specific spatial targeting within the CNS.

## Limitations

Although we saw increases in immediate-early gene promoter driven reporter expression with DBS, the increase was modest, and only occurred with repeated stimulation of the MSN. In addition, a potential source of variability in our experiment is the heterogenous viral tropism and infectivity across different regions of the hippocampus. This limited the extent of our spatial analysis to a predefined region within the DG immediately surrounding the injection site (Figure 3). Consistent with other reports (Taymans et al., 2007; Aschauer et al., 2013) viral tropism of the AAV serotype 5 is most robust and homogenous at the injection site, with non-linear and heterogenous spread into overlying and synaptically-connected regions. Finally, baseline expression of c-Fos by transduced neurons in the dentate gyrus during the 2-week period between viral injection and electrode implantation may have further masked the effect of DBS-inducible reporter activation.

We only tested two physiologically-relevant frequencies to the septohippocampal circuit: theta and gamma, which significantly enhanced TdT reporter expression. The mechanisms of DBS remain poorly understood and thus it remains possible that hippocampal oscillatory activity was affected with the first stimulation paradigm but did not sufficiently activate the c-Fos driven reporter to allow for detection (Jakobs et al., 2019; McKinnon et al., 2019).

It remains to be determined whether all frequencies of stimulation have this effect or whether there is the possibility to tune the frequency within the stimulated circuit. Future work will examine whether frequency can modulate reporter gene expression within the circuit and how this modulation may be correlated with altered changes of oscillatory activity in the stimulated circuit. Importantly, in our proof-of-concept study stimulation parameters in the gamma range which are currently routinely used in the clinical setting could induce expression of the reporter gene.

## Conclusion

Taken together, these data suggest that DBS can be used to regulate gene expression both spatially and temporally. Spatial regulation may be achieved by the site of injection and tropism of the AAV and temporal regulation may be modulated by stimulation parameters. In this study, the fluorescent TdT protein was used to detect and quantify viral-vector mediated reporter gene expression in the same circuit distal from the stimulated MSN target. Instead of fluorescent reporters, future gene therapy studies could utilize AAV expression with proteins promoting neuronal survival, such as brain-derived neurotrophic factor, or neurotransmitter-modulating genes, such as enzymes that enhance dopamine levels or change neuronal phenotypes from excitatory to inhibitory (McKinnon et al., 2019). Further investigation into other stimulation responsive promoters and stimulation parameters for successful control of gene expression by DBS for use in clinical applications such as neurodegenerative disease and movement disorders is warranted.

## Data Availability Statement

The datasets generated for this study are available on request to the corresponding author.

## Ethics Statement

The animal study was reviewed and approved by Animal Resources Centre – University Health Network.

## Author Contributions

AF, DL, CM, and SK contributed to the conception and design of the study. AF, DL, and CM performed surgeries. AF, EG, CM, MS, CN, and EL contributed to the histological preparation. AF wrote the first draft of the manuscript and performed statistical analysis. DL and SK wrote sections of the manuscript. CH, AL, and LK revised the manuscript and suggested additional experiments. All authors contributed to manuscript revision, read and approved the submitted version.

## Conflict of Interest

The authors declare that the research was conducted in the absence of any commercial or financial relationships that could be construed as a potential conflict of interest.
